# Chromosome-level genome assembly of Przevalski’s partridge (*Alectoris magna*)

**DOI:** 10.1038/s41597-023-02655-5

**Published:** 2023-11-25

**Authors:** Xumin Wang, Wenhao Xia, Xindong Teng, Wanying Lin, Zhikai Xing, Shuang Wang, Xiumei Liu, Jiangyong Qu, Wei Zhao, Lijun Wang

**Affiliations:** 1 College of Life Science, Yantai University, Yantai, Shandong 264005 China; 2 Qingdao International Travel Healthcare Center, Qingdao, Shandong 266071 China; 3 College of Life Science, Lanzhou University, No.222 Tianshui South Road, Lanzhou, 730000 Gansu China

**Keywords:** Genome, Comparative genomics, Bioinformatics

## Abstract

Przevalski’s partridge (*Alectoris magna*) is one of the birds in the genus *Alectoris* endemic to China. The distribution of *A. magna* was narrow, and it was only found in parts of the Qinghai, Gansu, and Ningxia provinces. *A. magna* was considered a monotypic species until it was distinguished into two subspecies. However, external morphological characteristics, rather than genetic differences or evolutionary relationships, are now commonly used as evidence of subspecies differentiation. In this study, a chromosome-level reference genome of *A. magna* has been constructed by combining Illumina, PacBio and Hi-C sequencing data. The 1135.01 Mb *A. magna* genome was ultimately assembled. The genome showed 96.9% completeness (BUSCO), with a contig N50 length of 23.34 Mb. The contigs were clustered and oriented on 20 chromosomes, covering approximately 99.96% of the genome assembly. Additionally, altogether 19,103 protein-coding genes were predicted, of which 95.10% were functionally annotated. This high-quality genome assembly could serve as a valuable genomic resource for future research on the functional genomics, genetic protection, and interspecific hybridization of *A. magna*.

## Background & Summary

Birds of the genus *Alectoris* are currently divided into seven species in total, Most of them are extensively distributed in Eurasia, and the subspecies diverge widely. Specifically, they are distributed as far east as the northern coast of China, as far north as southern Russia, and as far south as the Arabian Peninsula and Mediterranean islands^1,2^, and they were later introduced to Britain and the United States^3,4^.

*A. magna* is one of seven species in the genus *Alectoris*^5^ and is endemic to China. Przevalski’s partridge (*Alectoris magna*), which belongs to the family Phasianidae and genus *Alectoris*, is distributed only in the Qinghai, Gansu, and Ningxia provinces of China. Therefore, the distribution area is relatively narrow. Nevertheless, few studies have been conducted on *A. magna* in China. Large areas of land are presently being reclaimed for farmland in the already narrow distribution area of *A. magna*, while habitat conditions are deteriorating because of overhunting and the development of agriculture and animal husbandry^6,7^. In 2021, *A. magna* was listed on the second level of the Chinese List of National Key Protected Wildlife. The two subspecies of *A. magna* diverged about 500,000 years ago, there are significant differences in sequence variation between them, no shared haplotype and lack gene flow. A complete assembled genome would contribute to refining the reference criteria for subspecies differentiation. According to research, there is an asymmetric introgression between the two kinds of partridges (*Alectoris magna* and *Alectoris chukar*), which makes it difficult to correctly identify the species based only on morphology and also affects the genetic integrity of the existing species^8–10^. The resulting hybrids presented the characteristic of *A. magna* in morphology, nevertheless, it had a genotype similar to that of A. chukar. It was speculated that the genes of A. chukar might have flowed into the gene pool of *A. magna*, which would interfere with sampling and sequencing. Previously, the complete mitochondrial genome of the mountain chukar was determined, providing basic data for genetic research on this endangered species^6^. Currently, whole-genome data and resources, can provide a foundation for following researches on the origin, subspecies division, population dynamics, and genetic conservation of *A. magna*.

In this study, a high-quality chromosome-level genome of Przevalski’s partridge was generated by integrating PacBio HiFi, Illumina paired-end sequencing, and high-throughput chromatin conformation capture (HiC) technology. The final combined *A. magna* genome had an N50 contig length of 23.34 Mb. A total of 19,103 protein-coding genes were predicted, of which 95.10% were functionally annotated. The reference genome acquired in this study may serve as a valuable resource for future research on *A. magna*.

## Methods

### Sampling and sequencing

An adult specimens of *A. magna* was originally selected from Lanzhou, China. Blood obtained through jugular vein sampling were used for DNA extraction as well as genome sequencing and assembly. All the blood samples were freshly frozen and stored in liquid nitrogen until they were used for DNA extraction. The animal used in this study was reviewed and ratified by the Experimental Animal Welfare and Ethics Review Committee of Yantai University, Shandong, China.

Following the manufacturer’s protocols, whole genomic DNA was extracted by means of an E.Z.N.A. ® Blood DNA kit (OMEGA, USA), and sequencing libraries were made utilizing the Truseq Nano DNA Sample Preparation Kit (Illumina, USA). The resulting libraries with an insertion size of 450 bp were quantified using a TBS-380 Miniature fluorometer Picogreen (Invitrogen), sequenced on an Illumina NovaSeq6000 sequencing platform, and produced paired-end reads of 150 bp. Following Illumina sequencing, 66 Gb of raw genomic data for *A. magna* were obtained (Table 1). Subsequently, quality clipping of the raw data was performed to remove low-quality data and make the subsequent assembly more accurate. The base distribution and mass fluctuation of each circle for all sequencing reads were statistically analyzed using bioinformatics. As shown in the Illumina raw data quality control chart, the sequencing quality of the samples and library construction quality are directly reflected.

**Table 1:** Next generation sequencing data used for the genome *A. magna* assembly.

Libraries types	Inter size (bp)	Raw data (Mb)	Clean data (Mb)	Q20 (%)	Q30 (%)	GC content (%)
Illumina reads	450	66624.9	66251.3	97.58	95.25	41.81
Hi-C reads	450	114494.3	113617.9	97.14	94.30	41.55

After the library construction was complete, HiFi sequencing was performed using PacBio Sequel II. After processing the original data through a series of filters, 34.2 Gb reads with an average length of 14.2 kb passed quality control

To perform chromosome-level genome assembly, a Hi-C library was constructed utilizing the MboI restriction enzyme with a previously described standard protocol^11,12^. Briefly, after grinding the samples with liquid nitrogen, the cells were treated with formaldehyde to cross-link DNA with proteins. The crosslinked DNA was treated with restriction enzymes to generate sticky ends. The ends were then repaired, and biotin was introduced to label the oligonucleotide ends, which were subsequently ligated with T4 DNA Ligase. Protease digestion was used to remove the cross-linked state, and the purified DNA was broken into fragments 500–700 bp in length. The labeled DNA was captured using streptavidin magnetic beads. The Hi-C libraries were quantified and sequenced on an Illumina NovaSeq 6000, and sequencing data were applied in chromosome-level assembly^13^.

### Genome size estimation and ***de novo*** assembly of *A. magna*

**Fig. 1:**
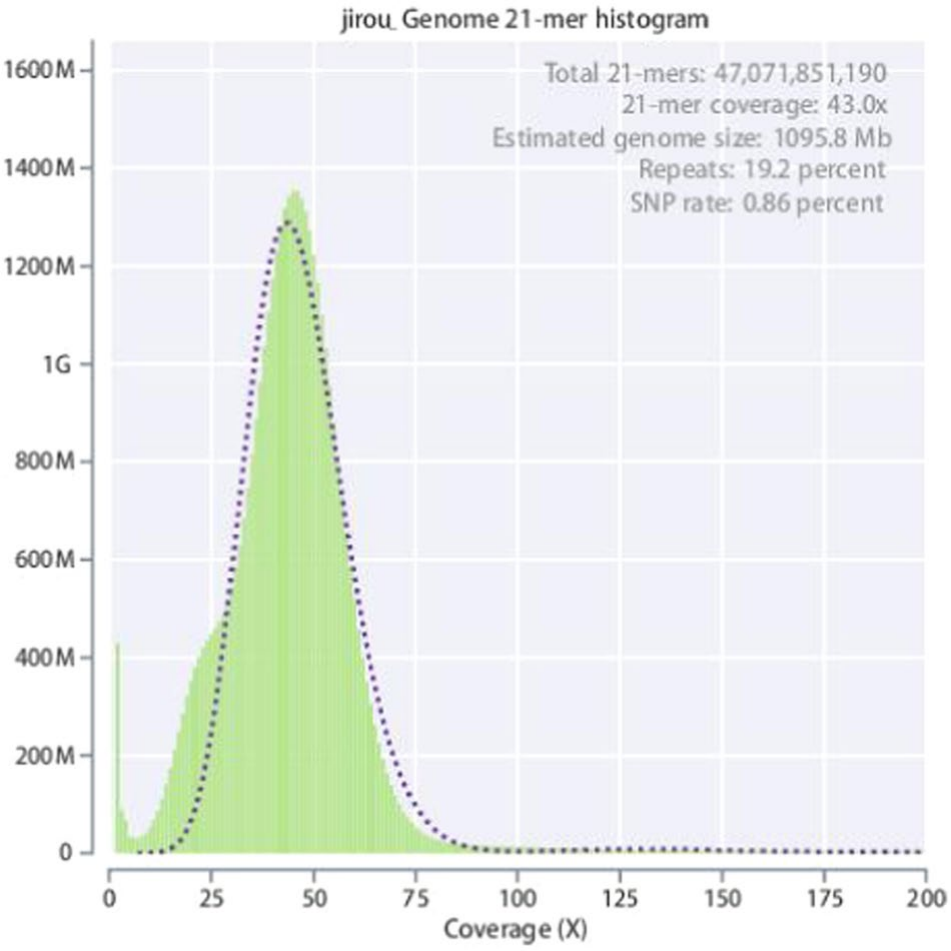
21-mer frequency distribution in *A. magna* genome.

Before genome assembly, analysis, and annotation, we used the K-mer statistics method to estimate genome size based on Illumina sequencing data. The K-mer size was set to 21 to analyze the data and estimate the genome size, heterozygosity, and repetition rate of the obtained samples^14^. On the basis of a total of 47,071,851,190 21-mers, the genome size was predicted to be 1095.8 Mb; meanwhile, the estimated heterozygosity and repeat rate were approximately 0.86% and 19.2%, respectively (Table 2 and Fig. 1).

**Table 2:** Evaluation of K-mer genome complexity.

Kmer	N Kmer	Genome size (Mb)	Heterozygousrate (%)	Repeatrate (%)
21	47,071,851,190	1095.8	0.86	19.2

PacBio HiFi long reads obtained by sequencing were preliminarily assembled using the HiFi data assembly software Hifiasm (https://github.com/chhylp123/hifiasm). Although the accuracy was high for the HiFi reads, some errors remained. Hifiasm reads all HiFi reads into memory for all-vs.-all alignment and error correction. Based on overlapping information between reads, if there is a base on the read that is different from other bases and it is supported by at least three reads, it is considered an Single Nucleotide Polymorphism (SNP) and retained; otherwise, it will be regarded as an error and corrected. Eventually, the long-read SMRTbell library^15^ yielded a genome assembly of 1135.01 Mb with a contig N50 of 23.34 Mb, which is similar to the results predicted by K-mer analysis.

### Chromosome-level genome assembly and assessment of the genome assemblies

**Fig. 2:**
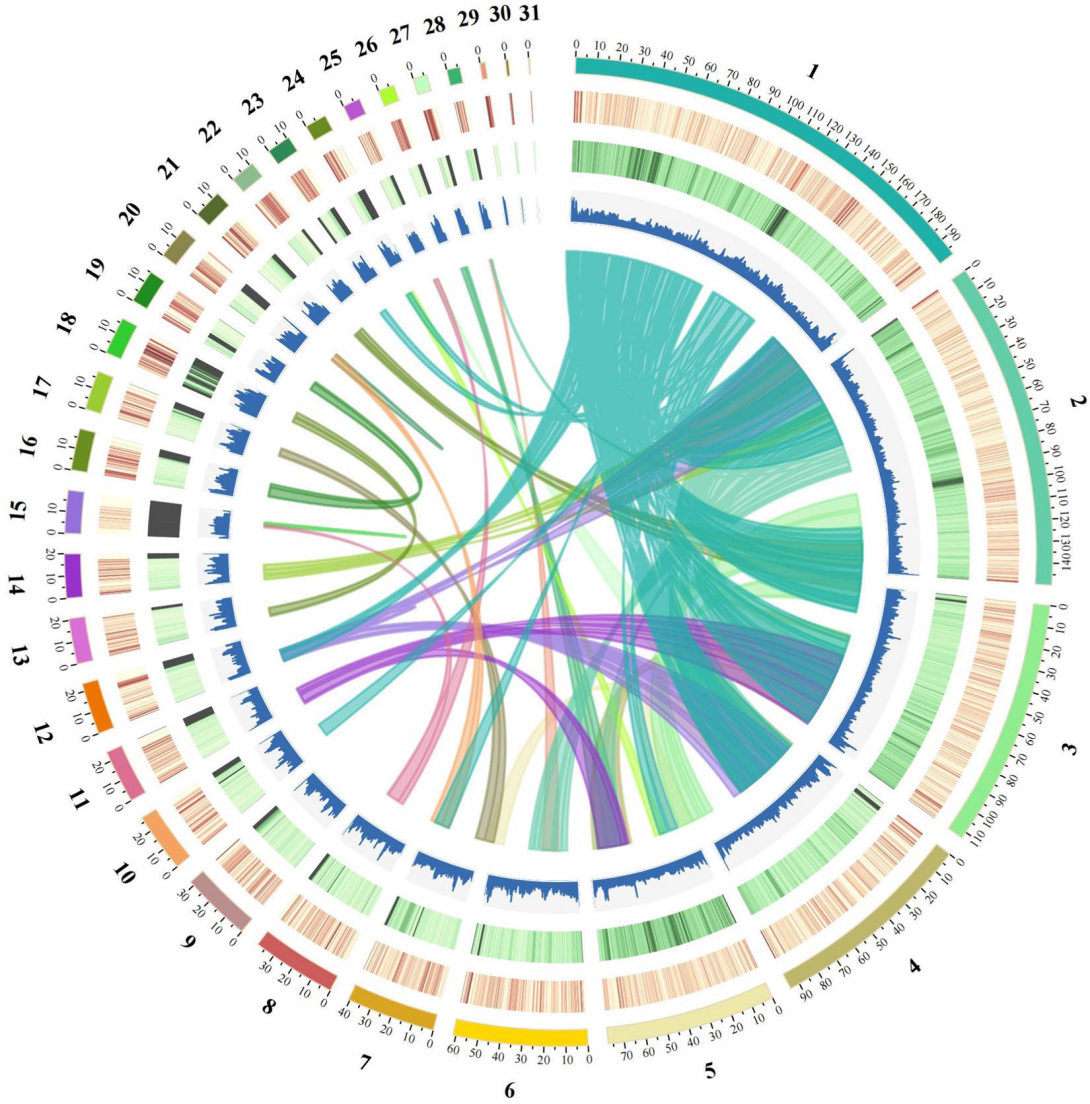
Genome Circos plot of *A. magna*. From the inner to the outer layers: Collinear gene blocks obtained by comparing genomes using MCScanX, GC content (100 kb window), percentage of repeats (100 kb window), gene density (100 kb window), Circular representation of the pseudomolecule.

**Fig. 3:**
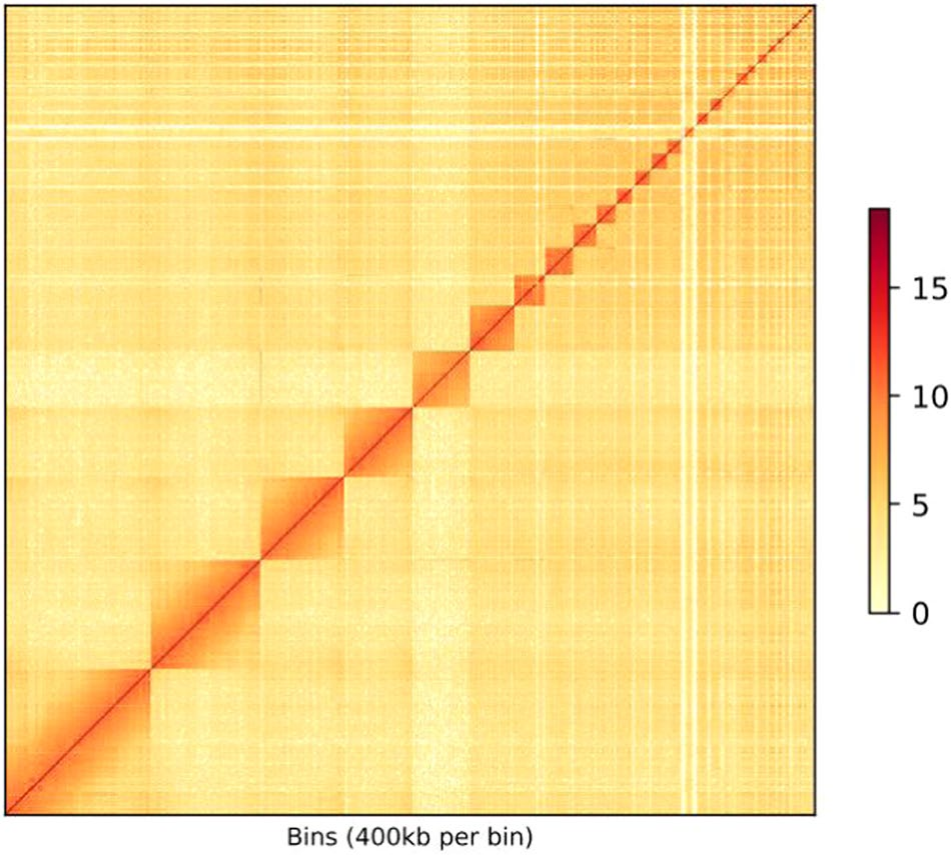
Hi-C assembly of chromosome interactive heat map.

Hi-C-assisted genome assembly was performed using Hi-C scaffolding methods^16^. Contigs from the previous assembly were clustered, and oriented toward the chromosome scale of the assembly. In total, 113.62 Gb of clean data were yielded from the Hi-C library (Table 1). Because the *cis* interaction was greater than the *trans* interaction, the Hi-C-corrected contigs were clustered, oriented, and anchored using an Allhic pipeline^17^. The final 1102.93 Mb (97.17%) assembled genome sequences were anchored on 31 chromosomes, with a chromosome length that ranged from 0.49 Mb to 198.20 Mb (Fig. 2 and Table 3). Additionally, the heat map of the Hi-C assembly interaction cassette was consistent with high-quality genome assembly (Fig. 3).

**Table 3:** Statistics of assembled chromosomes sequence length.

Sequences ID	Sequences Length	Sequences ID	Sequences Length
Chr1	198,201,711	Chr17	17,802,300
Chr2	149,776,179	Chr18	17,142,809
Chr3	113,072,824	Chr19	15,299,958
Chr4	94,451,134	Chr20	14,921,416
Chr5	77,158,717	Chr21	13,263,624
Chr6	61,304,909	Chr22	11,362,314
Chr7	40,627,020	Chr23	11,043,038
Chr8	38,852,039	Chr24	9,956,855
Chr9	32,053,122	Chr25	8,044,485
Chr10	27,096,778	Chr26	7,370,171
Chr11	24,420,583	Chr27	6,465,074
Chr12	23,540,526	Chr28	6,112,204
Chr13	20,824,369	Chr29	2,383,849
Chr14	20,023,070	Chr30	1,252,667
Chr15	19,562,803	Chr31	489,616
Chr16	19,060,355	—	—
Total	1,102,936,519	Percentage	97.17%

**Fig. 4:**
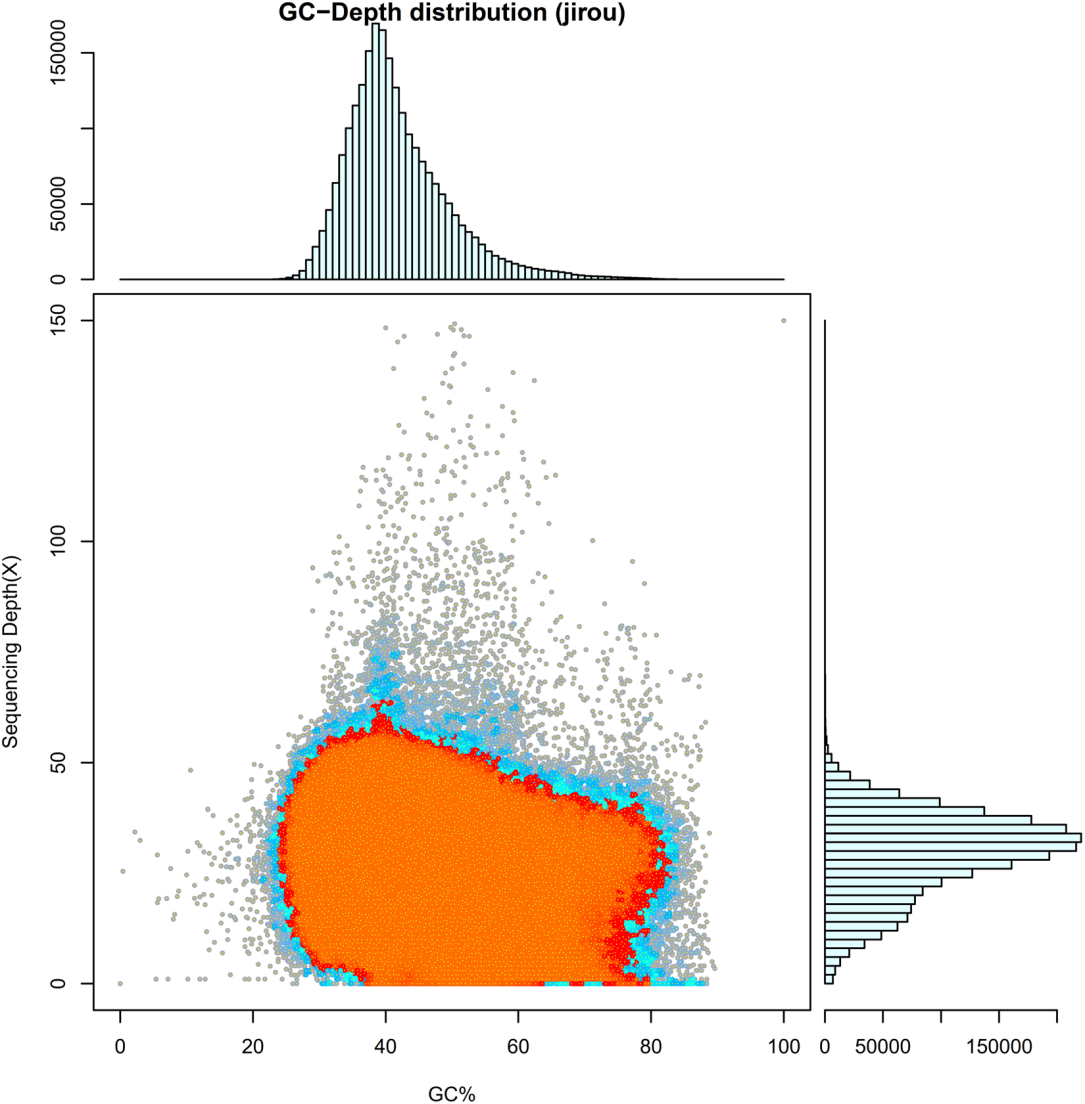
Statistical graph of correlation analysis between GC content and sequencing Depth. The abscissa represents GC content, the ordinate represents sequencing depth, the right is sequencing depth distribution, and the upper is GC content distribution.

GC-Depth was used to evaluate the assembly results and determine whether there was a significant GC bias or sample contamination^18^. The reads were aligned to the assembled sequences, both the GC content of the sequences and coverage depth of the reads were measured^19^. Following this, a correlation analysis was performed between GC content and sequencing depth (Fig. 4). In addition, the completeness of the assembly was assessed using Benchmarking Universal Single-Copy Orthologs (BUSCO v4.2.1)^20,21^ with the vetebrata_odb10 database and CEGMA^22^ software. The results showed that 96.9% (single-copy genes: 96.6%, duplicated genes: 0.3%) of the 8338 single-copy genes were identified as complete, 0.6% were fragmented, and 2.5% were missing from the assembled genome (Table 4). We also gained the integrity of the genome for 91.08% using merqury and the QV value and error rate of the genome obtained were 64.2452 and 3.76251e-07, respectively. In summary, these assessment results indicated that the *A. magna* genome assembly was of high quality.

**Table 4:** Results of the BUSCO assessment of *A. magna*.

Type	Number	Percentage (%)
Complete BUSCOs (C)	8085	96.9%
Complete and single-copy BUSCOs (S)	8058	96.6%
Complete and duplicated BUSCOs (D)	27	0.3%
Fragmented BUSCOs (F)	46	0.6%
Missing BUSCOs (M)	207	2.5%
Total BUSCO groups searched	8338	—

### Repetitive and non-coding gene prediction

Before predicting and annotating the protein-coding genes, repetitive elements in the *A. magna* genome were estimated through a combination of homologous comparison and ab initio prediction. The RepeatMasker (https://www.repeatmasker.org/) and Tandem Repeats Finder (https://tandem.bu.edu/trf/trf.html) software were used to identify scattered repeats and search for tandem repeats, respectively. Using RepeatMasker^23,24^, stray repeats were searched for by aligning the sequence with a database of known repeats (RepBase)^25,26^. Ultimately, we identified 361.2 Mb of repetitive sequences, including 229.1 Mb of interspersed repeats and 132.1 Mb of tandem repeats, accounting for 31.8% of the assembled genome. Among classified interspersed repeats, long interspersed repeated sequences (LINEs) were the most abundant with a whole length of 82 Mb, whereas rolling circle (RC) were the rarest with a total length of 0.67 Mb, which occupied 0.06% of the whole genome sequences (Table 5).

**Table 5:** Repeat elements in *A. magna* genome.

Repeats elements	Number	Total Length (bp)	In Genome (%)
**Interspersed repeats**			
LTR	98,926	33,355,672	2.9387
DNA	87,551	13,804,815	1.2163
LINE	267,470	82,021,435	7.2264
SINE	7,059	760,329	0.067
RC	4,337	674,128	0.0594
scRNA	0	0	0
Unknow	50,826	101,221,873	8.918
Subtotal	516,169	229,114,904	20.1858
**Tandem repeats**			
TRF	404,438	106,675,327	9.3985
Minisatellite DNA	304,063	21,953,263	1.9342
Microsatellite DNA	36,698	3,475,195	0.3062
Subtotal	745,199	132,103,785	11.6389
Total	1,777,537	361,218,689	31.8247

Region and secondary structure of the tRNAs were predicted using tRNAscan-SE v2.0.7^27^, and BLAST was used to predict the rRNA sequences. A total of 283 tRNAs were predicted using tRNAscan-SE, and 99 rRNA genes were annotated using BLASTN^28^. Beyond that, the prediction principles for the other three ncRNAs including sRNA, snRNA, and miRNA were similar. First, the Rfam software was utilized to compare and annotate the Rfam database^29^, and then its cmsearch program with default parameters was used to determine the final sRNA, snRNA, and miRNA.

### Protein-coding genes prediction and annotation

The protein-coding genes in the *A. magna* genome assembly were estimated using a combination of *de novo* prediction, homologous protein alignment, and transcriptome-based methods. Augustus v3.23^30^ was used for de novo prediction, and we downloaded the protein sequence of *Coturnix japonica* (GCF_001577835.2) from NCBI database and used TblastN v2.2.26 with an e-value of 1e^−5^ to align the protein sequence to the sample genome^31^. Then, to get an accurate spliced alignment, matching proteins were aligned to homologous genome sequences using GeneWise v2.4.1^32^, which was subsequently used for identification of the gene coding and intron regions. For RNA-Seq prediction, RNA sequencing data derived from blood samples were aligned to the *A. magna* genome fasta by TopHat v2.1.1 with default parameter^33,34^, and the alignment results served as inputs for Cufflinks v2.2.1 to predict the gene structure^35–37^. Transcriptome data were concatenated with Trinity v2.11.0 to obtain transcripts^38^. Subsequently, EvidenceModeler v1.1.1 was used to integrate these gene sets to obtain the coding genes of the sample genome^39^. As a result, 19,103 protein-coding genes were estimated with a mean Coding sequence (CDS) length of 1561 bp.

**Fig. 5:**
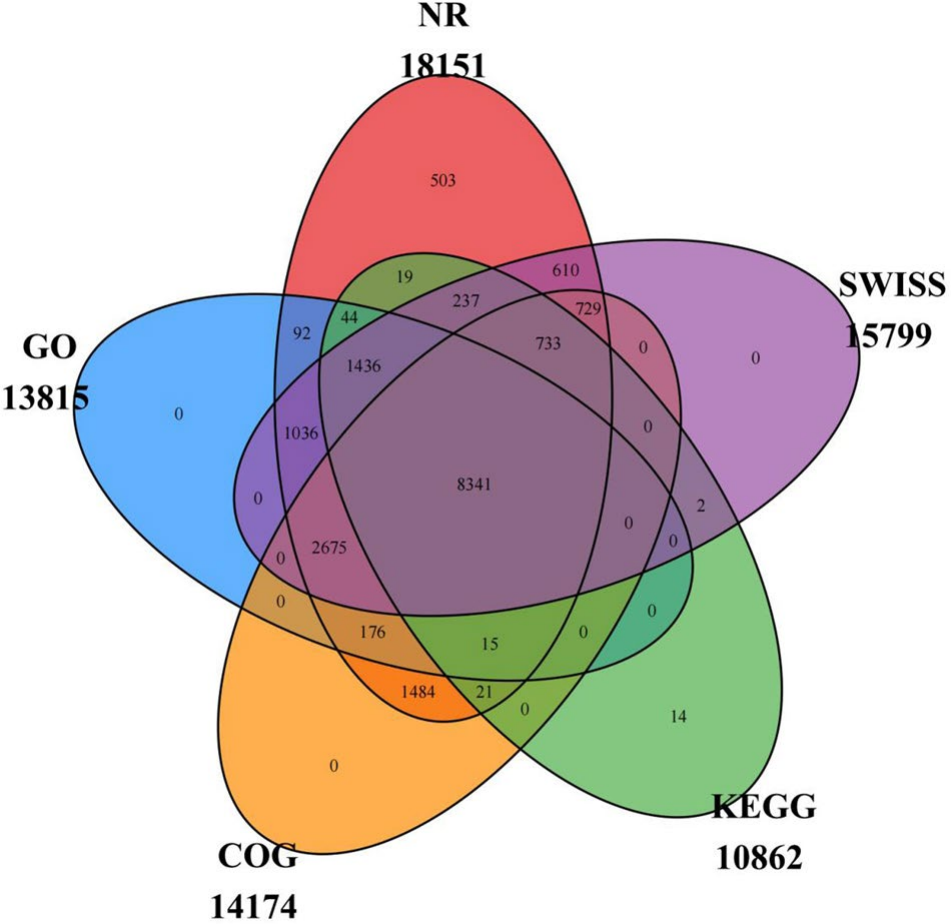
Venn diagram of the number of genes with functional annotation using multiple public databases.

The protein sequences of the predicted genes were compared with public biological functional databases, including the Nr, SwissProt^40,41^, GO^42^, eggNOG, and KEGG databases^43,44^, by blastp (BLAST + 2.7.1, comparison standard: e-value no more than 1e^−5^)^37^, and functional annotation was performed. Finally, a total of 18,167 genes were successfully annotated using at least one public database, representing 95.1% of the full of predicted genome (Table 6 and Fig. 5).

**Table 6:** Function annotation of genes by multiple methods.

Type	Number	Percentage (%)
Total	19103	100
NR	18151	95.02
GO	13815	72.32
COG	14174	74.2
KEGG	10862	56.86
SWISS	15799	82.7
In_all_DB	8341	43.66
AT_least_one_DB	18167	95.1

## Data Records

The whole-genome sequencing data (Illumina genomic sequencing reads, PacBio long reads, Hi-C data, and RNA-seq reads) were deposited in the National Center for Biotechnology Information (NCBI) Sequenced Read Archive (SRA) database at NCBI SRR23875790^45^, SRR23875789^46^, SRR23875788^47^, and SRR25722164^48^. The assembly genome was deposited at DDBJ/ENA/GenBank under the accession JARUNP000000000^49^. The assembly genome data, repeat sequence prediction and functional annotation results had been stored at Figshare^50^.

## Technical Validation

### Data filtering and quality control

Fast QC v0.11.8 was used to determine the quality of the sequences in the initial sequencing data. The original sequencing data contained low-quality reads, high N content, and contaminated adapters. In order to improve the accuracy of the subsequent assembly, Trimmomatic v0.39^51^ software was used to eliminate these; the specific steps included removing the adapter sequence from reads, pruning the read ends with lower sequencing quality (with a sequencing mass value less than 20), and removing reads containing more than 10% N bases. Eventually, we obtained clean reads stored in the fastq format.

### Assembly validation

To ensure the accuracy and continuity of the genome for subsequent annotation and comparative genome analysis, the integrity of the genome assembly must be accurately evaluated after its completion. Three genomic quality assessments were used to comprehensively detect the genome assembly: sequencing depth/coverage, GC distribution, Merqury, and BUSCO assessments. The GC content distribution and sequencing coverage of an assembled sequence were determine based on a GC depth distribution map. Merqury evaluates the genome based on Kmer to obtain consistency quality (QV), genome assembly error and completeness. BUSCO assessment compares homologous genes in the genome assembly results to predict the integrity of the gene regions of the genome assembly, especially conserved gene regions.

## Data Availability

If no detailed parameters were mentioned, all software and tools in this study were used with their default parameters. No specific code or script was used in the study.
